# VIEWPOINT FROM THE DIVISION OF GERIATRICS AND CLINICAL GERONTOLOGY

**DOI:** 10.1093/geroni/igz038.1642

**Published:** 2019-11-08

**Authors:** Evan Hadley

**Affiliations:** National Institute on Aging, National Institutes of Health, Bethesda, Maryland, United States

## Abstract

Dr. Hadley will review accomplishments of the Division of Geriatrics and Clinical Gerontology and make projections for the future.

